# Genetic deletion of *ITIH5* leads to increased development of adipose tissue in mice

**DOI:** 10.1186/s40659-024-00530-0

**Published:** 2024-08-29

**Authors:** Thomas M. Sessler, Justus P. Beier, Sophia Villwock, Danny Jonigk, Edgar Dahl, Tim Ruhl

**Affiliations:** 1 Department of Plastic Surgery, Hand Surgery-Burn Center, University Hospital RWTH Aachen, Pauwelsstraße 30, 52074 Aachen, Germany; 2 Institute of Pathology, University Hospital RWTH Aachen, Pauwelsstraße 30, 52074 Aachen, Germany; 3 Biomedical Research in End-stage and Obstructive Lung Disease Hannover (BREATH) of the German Center for Lung Research (DZL), Hanover, Germany

**Keywords:** Adipokine, Adipogenesis, Adiposity, Inflammation, Homeostasis

## Abstract

**Background:**

Adipocytokines play a pivotal role in maintaining adipose tissue homeostasis by regulating cellular metabolism, proliferation, differentiation, and secretory activity. These soluble factors are relevant components for healthy adipose tissue, while their deficiency is closely associated with the development of obesity and related metabolic diseases, e.g., chronic inflammation. In human adipose tissue, inter-α-trypsin inhibitor heavy chain 5 (ITIH5) is expressed in proportion to the development of adipose tissue, i.e., the individual’s BMI. Thus, ITIH5 has been proposed to be an inert marker of human obesity. However, when applied to adipose stem cells in vitro, recombinant (r)ITIH5 protein inhibited proliferation and adipogenesis, suggesting that ITIH5 negatively affects the development of fat mass. We now tested the role of ITIH5 in vivo and compared *ITIH5*^*+/+*^ wildtype with *ITIH5*^*−/−*^ knockout mice.

**Results:**

Genetic deletion of *ITIH5* significantly increased adipose tissue mass relative to animal bodyweight (*p* < 0.05). Next, we characterized adipose stem cells (ASCs) from both genotypes in vitro. *ITIH5*^*−/−*^ cells exhibited increased proliferation and adipogenic differentiation (*p* < 0.001), which could explain the increase in adipose tissue in vivo. Furthermore, ASCs from *ITIH5*^*−/−*^ animals were more responsive to stimulation with inflammatory mediators, i.e., these cells released greater amounts of IL-6 and MCP-1 (*p* < 0.001). Importantly, the application of the rITIH5 protein reversed the observed knockout effects in ASCs.

**Conclusions:**

Our data suggest that ITIH5 potently regulates adipose tissue development and homeostasis by modulating ASC biology in mice. In addition, the effect of the rITIH5 protein underscores its potential as a therapeutic agent to correct the adipose tissue dysregulation often associated with obesity and metabolic disorders.

**Supplementary Information:**

The online version contains supplementary material available at 10.1186/s40659-024-00530-0.

## Background

Adipose tissue is an important endocrine organ that secretes numerous soluble factors, e.g., hormones, growth factors and cytokines, which are referred to as adipocytokines or adipokines. These molecules mediate cell maintenance and tissue homeostasis by modulating physiological processes such as proliferation, metabolic and secretory activity, migration and differentiation [1]. Several adipokines are released into the bloodstream and act on distant organs and tissues. However, some adipokines have been classified based on their auto- and paracrine activity in adipose tissue.

Representatives of this group of adipokines include, for example, vaspin, chemerin and adiponectin. Vaspin (SERPIN A12) is increasingly released during adipose tissue development, which explains its high serum concentration in people with obesity, and it suppresses inflammation in adipose tissue [2]. Chemerin is released from adipose tissue and binds to the membrane receptor CMKLR1 (chemerin chemokine-like receptor 1) of adipocytes and their precursors, the adipose stem cells (ASCs), thus, regulating cell metabolism and differentiation [3]. Adiponectin is released in proportion to adipogenesis and acts inversely on adipocytes by stimulating beta-oxidation, inhibiting gluconeogenesis and increasing insulin sensitivity [4, 5]. Thus, adipokines are critically involved in the regular development and function of adipose tissue in health and disease [6].

In a recent study, we investigated the function of Inter-α-trypsin inhibitor heavy chain 5 (ITIH5) in human adipose tissue [7]. The inter-α-trypsin inhibitor family members are ancient molecules that have evolved during the evolution of vertebrates several hundred million years ago [8, 9]. These molecules consist of bikunin-linked proteoglycans with heavy chain proteins covalently bound to chondroitin sulfate [10]. Apart from their general role in stabilizing the extracellular matrix (ECM), these molecules interact with other components of the ECM, particularly hyaluronic acid, to regulate various cellular functions [11–14]. ITIH5 inhibits tumor growth and metastasis especially by modulating TGF-β signaling [15]. The antitumor activity of ITIH5 has been extensively studied in various tumor entities such as breast cancer, colon cancer and lung cancer [15–19]. In all of these tumor entities, clinical collectives have shown that ITIH5 is successively lost during tumor progression and that its loss is associated with unfavorable survival. It has been consistently shown that the cause of this loss of expression is primarily DNA hypermethylation of the *ITIH5* promoter in cancer cells [20–23]. However, it was also shown early on that ITIH5 is abundantly expressed in adipose tissue [24]. The concentration of ITIH5 in adipose tissue correlates with the patient’s BMI, implying that ITIH5 is increasingly secreted during adipogenesis [7]. In vitro, the application of recombinant (r)ITIH5 protein inhibits proliferation and particularly the adipogenic differentiation of ASCs [7]. Therefore, it is hypothesized that ITIH5 acts as a negative regulator of adipose tissue hyperplasia and hypertrophy in vivo. Furthermore, the ITIH5 protein exhibits immunosuppressive effects by downregulating the release of interleukin (IL)-6 and monocyte chemoattractant protein (MCP)-1, when ASCs are exposed to an artificial inflammatory environment [7]. Based on these findings, ITIH5 has been proposed as a potential novel target for controlling or modulating adipose tissue homeostasis to counteract the development of obesity and related metabolic disorders.

To validate our recent in vitro findings of ITIH5 in human cells and tissues in an animal model, we have now investigated the in vivo effects of the genetic deletion of *ITIH5* in mice. We analyzed and compared the body weight and fat pad weight of wildtype (*ITIH5*^*+/+*^) versus knockout (*ITIH5*^*−/−*^) animals at 8 weeks of age. We then isolated ASCs from adipose tissue of the animals and tested whether the *ITIH5*-knockout affects proliferation and the multipotent differentiation potential of the cells. Because cellular differentiation is generally affected by TGF-signaling, which is reported to be modulated by ITIH5, we analyzed adipogenesis, osteogenesis and chondrogenesis of ASCs to test for an overall effect of deleting *ITIH5*. Secretory activity was analyzed on cell supernatants using ELISA against IL-6 and MCP-1 after inflammatory stimulation. In the case that the knockout of *ITIH5* impaired ASC biology, we tested the compensatory effect of rITIH5 protein application. Our results provide novel insights into the physiological function of ITIH5 in mammalian adipose tissue and highlight the importance of studying the effects of adipokines in vivo.

## Methods

### Materials

From Santa Cruz (Heidelberg, Germany): Resazurin sodium salt. From Life Technologies (Darmstadt, Germany): Fetal bovine serum (FBS), Insulin-Transferrin-Selenium (ITS) premix, high/low glucose medium (4.5 g/l; 1 g/l) and Dulbecco’s Modified Eagle’s medium (DMEM/F-12). From Worthington Biochemical Corp. (Lakewood, NJ, USA): Collagenase (type I). From Sigma (Taufkirchen, Germany): Safranin O, rosiglitazone, ascorbate 2-phosphate, β-glycerophosphate, 3-Isobutyl-1-methylxanthine (IBMX), paraformaldehyde (PFA), trypsin–EDTA, penicillin–streptomycin, 2-amino-2-methyl-1-propanol (AMP), o-cresolphthalein complexon, 8-hydroxyquinoline, bovine serum albumin (BSA) and Tween 20. From Roth (Karlsruhe, Germany): l-Prolin, acetic acid, hydrochloric acid (HCl), crystal violet. From Roche (Mannheim, Germany): Protease inhibitor and insulin. From PeproTech (Hamburg, Germany): Basic fibroblast growth factor (bFGF), transforming growth factor (TGF)-β3, tumor necrosis factor (TNF)-α, interleukin (IL)-1β. From Merck (Darmstadt, Germany): HEPES, isopropyl alcohol, Oil Red O. From Otto Fischer (Saarbrücken, Germany): xylol. From Biochrom (Berlin, Germany): PBS.

### Tissue samples

Wild type (WT) mice (BALB/c) were obtained from the in-house breeding stock of the Institute of Laboratory Animal Science at the University Hospital, RWTH Aachen. *ITIH5*^*−/−*^ mice (same strain) were generated by TaconicArtemis (Cologne, Germany) as described previously [25]. At the age of 3 weeks, the offspring of each genotype was weaned and labeled by ear punch. The tissue was used for genotyping to determine *ITIH5*^*−/−*^ and *ITIH5*^*+/+*^ (WT). The mice were housed in groups of 5 animals in plastic cages with sawdust bedding and had access to food and water *ad libitum*. The animal housing rooms were kept at 21–24 °C and 40–60% relative humidity with a 12-h light/dark cycle. Mice were sacrificed for experimental investigation at the age of 8 weeks (*n* = 31 per genotype, both sexes). After cervical dislocation under deep anesthesia (5% isoflurane), the bodyweight of each animal was determined. The skin was disinfected with 70% ethanol, and adipose tissue was obtained from fully excised perigonadal and perirenal fat deposits. The animals were only used for organ removal for scientific purposes according to § 4 of the animal welfare act (TierSchG), which is not an animal experiment as subject for approval by the authority (LANUV). However, under the German Decree on the Reporting of Laboratory Animals (VersTierMeldV) the animals used were recorded, documented, and reported. A form with the internal number 40084A4 that includes the scientific purpose is documented at the Institute of Laboratory Animal Science at the University Hospital, RWTH Aachen.

### Adipose stem cell isolation

Isolation of mouse ASCs, cell expansion and differentiation experiments followed the same protocols as described previously [26]. After the whole adipose tissue explant were weighed, they were minced with surgical scissors and digested in 0.2% collagenase I (Worthington Biochemical Corporation, Lakewood, NJ) for 60 min at 37 °C. After filtering the digested tissue through a 250 nm nylon mesh (neolab, Heidelberg, Germany) and centrifuging at 400x g, the stromal vascular fraction was separated from the remaining fluid and tissue. The cell pellet was cultured in proliferation medium (DMEM supplemented with 0,1% bFGF, 10% FBS) under standard conditions. The experiments were performed with cells from P2-P5 seeded at a density of 20.000 cells per cm², unless otherwise indicated.

### Crystal violet assay

After washing with PBS and fixation with isopropyl alcohol for 10 min at RT, the cells were washed again with 0.05% Tween 20 in PBS. The cells were then stained with 0.1% crystal violet (CV) for 20 min. After careful removal of the staining solution and five washing steps with aqua_dest_, the absorbed dye was removed by washing out with 33% acetic acid with gentle shaking. The samples were pipetted at 70 µl in duplicate and the absorbance was measured on a microplate reader at 620 nm (BMG Labtech, Ortenberg, Germany).

### Adipogenic differentiation

For adipogenic differentiation, the cells were exposed to adipogenic differentiation medium (high glucose medium supplemented with 2% FBS, 4 µM insulin, 0.28 µM rosiglitazone, 1 µM dexamethasone, 20 µM IBMX) and cultivated for 14 d. The cells were then washed with PBS, fixed with PFA (20 min) and washed again with PBS. The cells were stained with 0.2% Oil Red O for 30 min. After three washes with aqua_dest_, the stained cells were washed with isopropyl alcohol for 15 min with gentle shaking. The absorbance of 100 µl of each sample was measured in triplicate at 540 nm.

### Osteogenic differentiation

The cells were exposed to osteogenic differentiation medium (low glucose medium supplemented with 10% FBS, 0.25 µM dexamethasone, 200 µM L-Ascorbic-Acid-2-PO_4_ and 10 mM β-glycerolphosphat) for 14 d. The cresolphthalein assay was used to quantify calcium deposition as an indicator of cellular osteogenesis. After washing with PBS, the cells were fixed with 4% PFA for 10 min. After washing with aqua_dest_, cresolphthalein buffer (50 mg o-cresolphthalein complexon and 500 mg 8-hydroxyquinoline dissolved in 30 ml of HCl (370 ml/l), diluted in 500 ml aqua_dest_) was added to the cells for 5 min, followed by incubation with AMP-Buffer (76 ml AMP in 500 ml aqua_dest_, pH = 10.7 with HCl) for 15 min. Absorbance was measured at 580 nm in 100 µl triplets.

### Chondrogenic differentiation

The cells were stimulated for 5 d with chondrogenic differentiation medium (low glucose medium supplemented with 1 µM dexamethasone, 0.17 mM L-ascorbic-acid-2-PO_4_, 350 µM L-prolin, 1% ITS, and 5 ng/ml TGF-β3). Safranin-O staining was performed to detect glycosaminoglycans as a measure of chondrogenic differentiation. The cells were washed with PBS and fixed with 4% PFA for 10 min. After washing with PBS, the cells were stained for 30 min with 0.1% Safranin O. The adsorbed dye was removed by washing with isopropyl alcohol for 20 min. The absorbance was measured at 540 nm in triplets of 100 µl.

### Cytokine determination by ELISA

To measure cytokine release, cells were seeded at a density of 2.000 per cm² [27]. Since the concentrations of IL-1β and TNF-α are increased in the adipose tissue of people with obesity but are not secreted by ASCs [28], these factors are suitable for mimicking an artificial inflammatory environment for the cells in vitro. The cells were incubated for 48 h with 1 ng/ml of IL-1β or TNFα, or of both. The supernatants were collected and the concentrations of IL-6 and MCP-1 were determined using ELISA Duo-Sets (R&D Systems, Minneapolis, MN, USA) following the manufacturer’s instructions. The results were normalized to the cell numbers assessed by CV staining as described above.

### Recombinant ITIH5 protein

In each case where the genetic knockout of *ITIH5* affected a cellular characteristic of ASCs, we treated the cells with recombinant protein to test for a compensatory effect. The recombinant ITIH5 protein used in this study (spanning 681 amino acids) reflects the secreted N-terminal region of ITIH5 including the VIT domain (Interpro number IPR013694) and the vWA domain (Interpro number SM00327). The cloning of the pMS-L-A-IV vector of rITIH5, the in vitro synthesis based on HEK293T cells and its functional, i.e., tumor suppressive, activity have already been described and confirmed [29]. Analogous to this procedure, rITIH5-derived proteins present in the supernatant of HEK293T cells were purified by using Ni-NTA agarose beads (Qiagen, Hilden, Germany) and re-buffered in PBS via Vivaspin 6 columns (Sartorius Stedim, Göttingen, Germany) according to the manufacturer’s instructions. Western blot analyses were used to confirm the purity and amount of the corresponding rITIH5 protein batches. Recombinant protein was applied at concentrations of 0.01 and 0.1 µg/ml for cell culture experiments based on our previous study on human ASCs [7], which revealed a significant impact on cell proliferation, differentiation, and the release of soluble factors.

### Analyses and statistics

The data of all experiments were grouped to assess the results for each type of experiment and treatment. The Kolmogorov-Smirnov test was used to test for normal distribution. Normally distributed data were presented as mean values (+ SEM) and were statistically analyzed by analysis of variance (one-way ANOVA) followed by the Tukey post-hoc test. Non-normally distributed data were presented as box-plots (median as the middle line, 25/75% as box boundaries and min/max as whiskers) and were statistically analyzed by the Kruskal-Wallis H-test followed by pairwise comparison using the Mann-Whitney U-test after Bonferroni procedure. The effect size was measured using Cohen’s d formula (SPSS 24, SPSS Inc., Chicago, USA). The calculated d-values of 0.2–0.49 were considered small, 0.5–0.79 were considered medium, and d > 0.8 was interpreted as a large effect size. Differences associated with *p* < 0.05 were considered statistically significant. Figures were created using Corel Draw X5 (Corel Corporation, Ottawa, Canada).

## Results

### *ITIH5* knockout increased relative fat mass

**Fig. 1:**
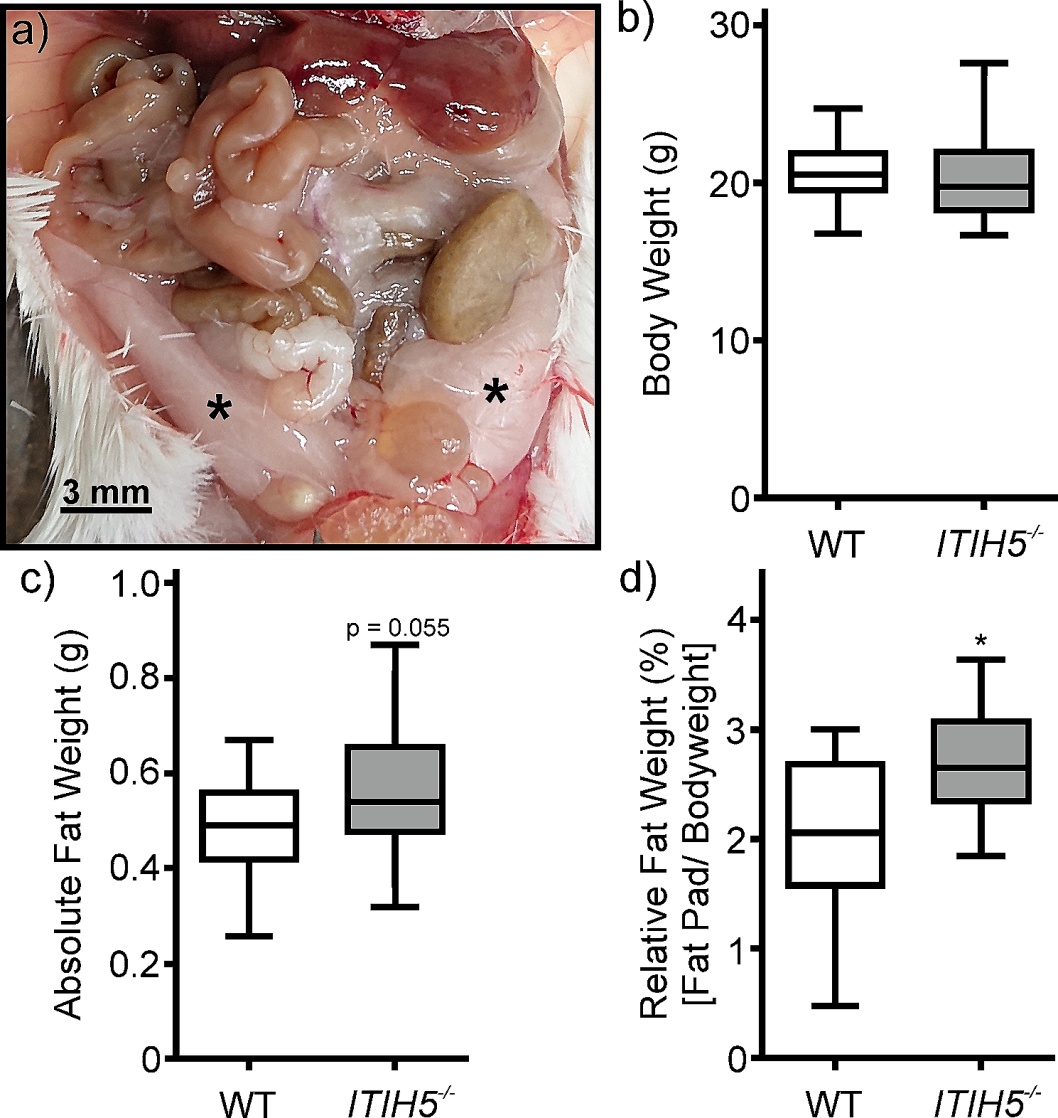
Gross anatomical location of **a**) gonadal adipose depots of the mouse (asterisks). Effect of genetic deletion of ITIH5 (*ITIH5*^*−/−*^) on **b**) bodyweight, **c**) absolute fat mass, and **d**) adipose tissue weight relative to bodyweight. Wildtype (WT) and knockout mice (*n* = 31 each) were compared at 8 weeks of age. Pairwise comparison was performed using Mann-Whitney U-test, *p < 0.05

To investigate the role of the ITIH5 protein in adipose tissue of mice, the body weight of WT and *ITIH5*^*−/−*^ animals and the absolute and relative masses of gonadal and perirenal fat pads were determined (Fig. 1a). The U-test revealed no difference in body weight between the two genotypes (U = 1.16, *p* = 0.245, d = 0.24; Fig. 1b). However, the absolute fat mass of the tissue explants tended to be greater in the *ITIH5*^*−/−*^ than in the WT animals (U = 1.92, *p* = 0.055, d = 0.54; Fig. 1c). The adipose tissue mass in relation to body weight was greater in the knockout mice (median = 2.86 g) than in the WT animals (median = 2.4 g). This difference was significant (U = 2.53, *p* = 0.012, d = 0.65; Fig. 1d).

### *ITIH5* knockout increased the proliferation of ASCs

**Fig. 2:**
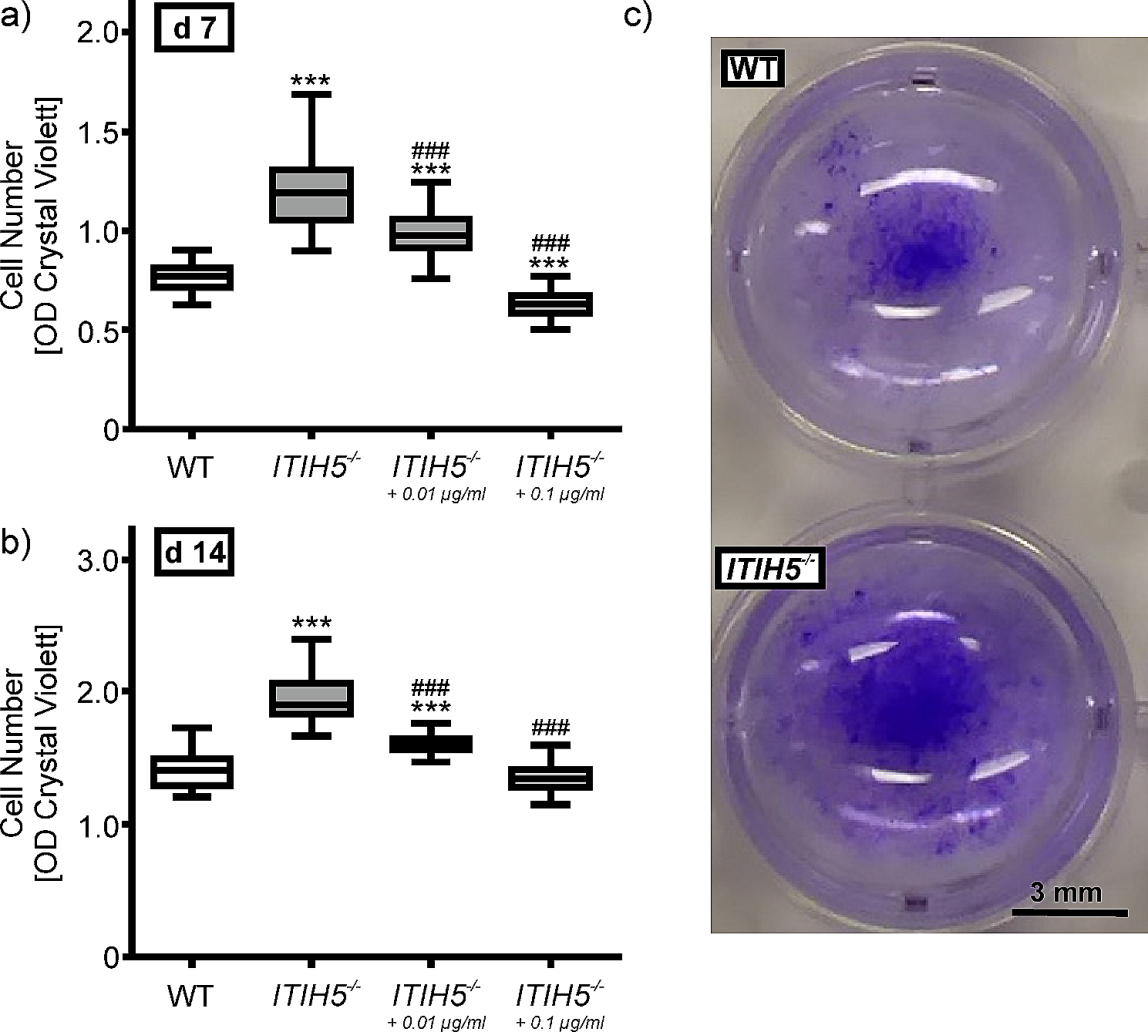
Effect of genetic deletion of ITIH5 (*ITIH5*^*−/−*^) on the proliferation of ASCs compared to the cells from wildtype (WT) animals (*n* = 5 each). *ITIH5*^*−/−*^ cells were either untreated or stimulated with 0.01 µg/ml or 0.1 µg/ml rITIH5 protein. The cell number was assessed by the optical density (OD) of crystal violet (CV) staining after (**a**) 7 days and (**b**) 14 days of incubation. Images in (**c**) indicate differences in CV staining between WT and *ITIH5*^*−/−*^ cells without stimulation with the rITIH5 protein. The number of experiments was *n* ≥ 87. Statistical analyses were performed by the Kruskal-Wallis H-test followed by pairwise comparisons using the Mann-Witney U-test after Bonferroni procedure; ***p < 0.001 vs. WT; ^###^*p* < 0.001 vs. *ITIH5*^*−/−*^

The Kruskal Wallis H-test revealed significant differences between the experimental groups after 7 d (H(3) = 296.278; *p* < 0.001; Fig. 2a), and after 14 d (H(3) = 280.034; *p* < 0.001; Fig. 2b), as determined by CV staining, which measures the number of ASCs (Fig. 2c). Genetic deletion of *ITIH5* resulted in a significantly greater cell number both after 7 d (*ITIH5*^*−/−*^ OD_median_=1.194; WT OD_median_=0.768; *p* < 0.001, d = 0.97) and after 14 d (*ITIH5*^*−/−*^ OD_median_=1.898; WT OD_median_=1.408; *p* < 0.001, d = 3.08). The Application of the rITIH5 protein induced a concentration-dependent reduction in CV staining, indicating that exposure to the rITIH5 protein compensates for the genetic knockout effect. Pairwise comparisons on day 7 revealed that rITIH5 protein at 0.01 µg/ml (OD_median_=0.971) and 0.1 µg/ml (OD_median_=0.629) significantly decreased proliferation in *ITIH5*^*−/−*^ cells (*p* < 0.001, d > 1.4). While proliferation was still increased at 0.01 µg/ml, the higher concentration of 0.1 µg/ml rITIH5 protein led to a significant reduction in cell number below the number in WT. The same effects also occurred after 14 d; exposure to the rITIH5 protein at 0.01 µg/ml (OD_median_=1.602) significantly reduced the knockout effect. Treatment with the rITIH5 protein at a concentration of 0.1 µg/ml (OD_median_=1.343) reduced the cell number as much as to the WT phenotype.

### *ITIH5* knockout reduced the differentiation of soft tissue in ASCs

**Fig. 3:**
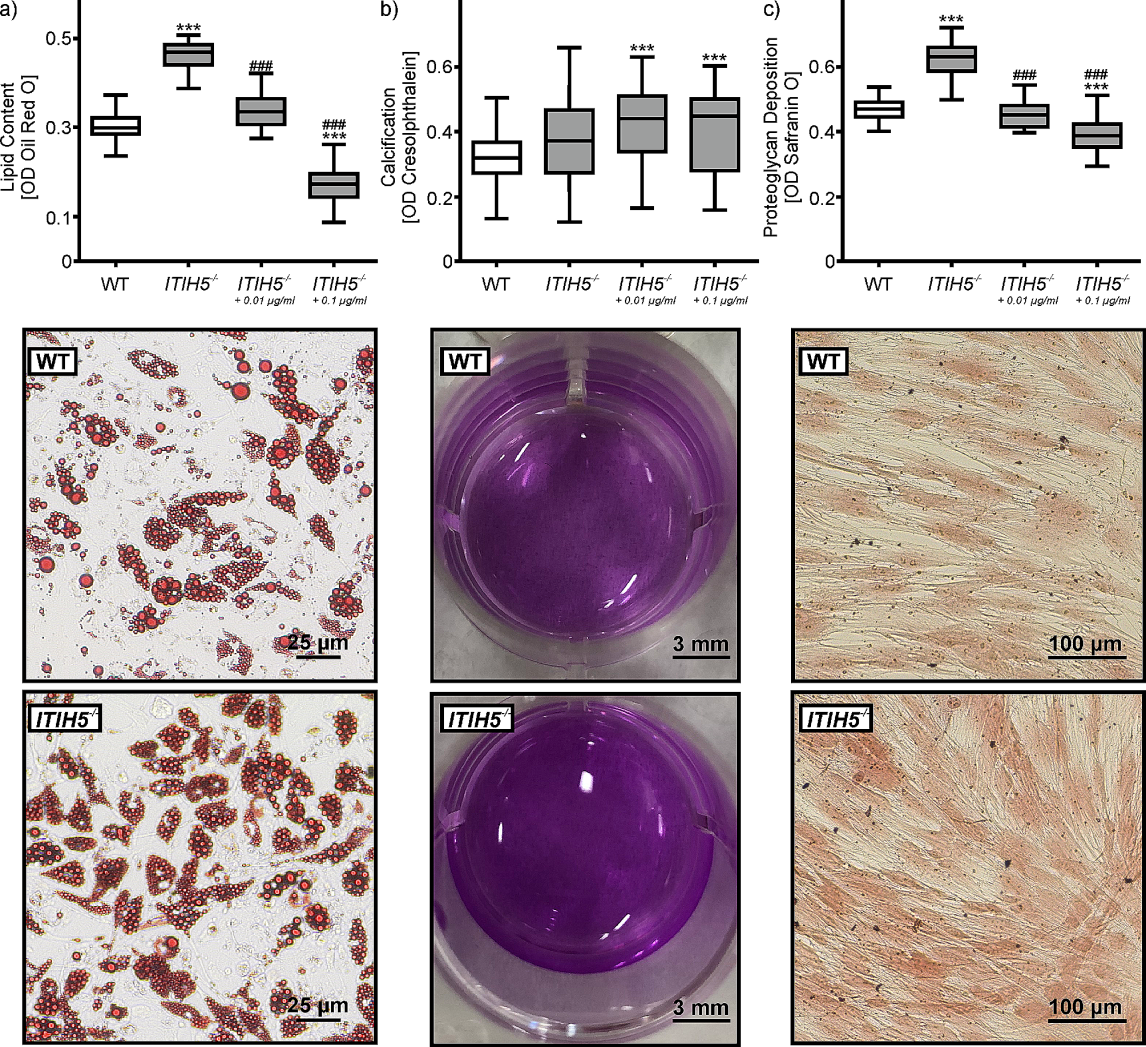
Effect of genetic deletion of ITIH5 (*ITIH5*^*−/−*^) on the trilinear differentiation of ASCs compared to cells from wildtype (WT) animals (*n* = 4 each). Adipogenesis was measured after 14 days by **a**) Oil Red O staining. Osteogenesis was analyzed by **b**) cresolphthalein staining for quantification of extracellular matrix calcification after a 14 day incubation period. After 5 days of stimulation, chondrogenesis was quantified by **c**) Safranin O staining. The photographs below each graph indicate differences in the number and size of stained lipid droplets of adipocytes, in cresolphthalein staining, and in proteoglycan formation of stained chondrocytes. The number of experiments was *n* = 68 for a), *n* ≥ 55 for **b**), and *n* ≥ 57 for **c**). Statistical analyses were performed by the Kruskal-Wallis H-test followed by pairwise comparisons using the Mann-Witney U-test after Bonferroni procedure; ***p < 0.001 vs. WT; ^###^*p* < 0.001 vs. *ITIH5*^*−/−*^

After 14 d of adipogenic differentiation, the H-test showed significant differences in Oil Red O staining between the experimental groups (H(3) = 233.550; *p* < 0.001). A pairwise comparison revealed that adipogenic differentiation was significantly lower in WT ASCs (OD_median_=0.298) than in *ITIH5*^*−/−*^ cells (OD_median_=0.469; *p* < 0.001, d = 4.71) (Fig. 3a). The application of the rITIH5 protein reduced the knockout effect. A pairwise comparison revealed that 0.01 µg/ml of rITIH5 (OD_median_=0.335) reversed the knockout effect to a level similar to that in the WT cells. Furthermore, the rITIH5 protein at 0.1 µg/ml (OD_median_=0.173) inhibited adipogenic differentiation compared to the WT cells (*p* < 0.001, d > 3.6).

The H-test also determined significant differences in calcium deposition after osteogenic differentiation (H(3) = 30.278; *p* < 0.001; Fig. 3b). Pairwise comparison revealed that the genetic deletion of *ITIH5* did not affect osteogenic differentiation (WT OD_median_=0.319; ITIH5^−/−^ OD_median_=0.372; *p* = 0.071, d = 0.52). The application of the rITIH5 protein to *ITIH5*^*−/−*^ cells significantly increased osteogenic differentiation compared to that in the WT cells (*p* < 0.001, d > 0.3).

There were also significant differences in chondrogenic differentiation between the experimental groups (H(3) = 141.050; *p* < 0.001). The *ITIH5* knockout cells showed the strongest Safranin O staining (OD_median_=1.169; Fig. 3c), which was significantly greater than that in the WT cells (OD_median_=0.868; *p* < 0.001, d = 3.29). Exposure to 0.01 µg/ml of the rITIH5 protein inhibited chondrogenic differentiation in the *ITIH5*^*−/−*^ cells to the same level as in the WT cells (OD_median_=0.832). rITIH5 protein at a concentration of 0.1 µg/ml reduced chondrogenic differentiation below that of WT cells (OD_median_=0.711). This difference was significant (*p* < 0.001, d > 2.7).

### *ITIH5* knockout enhanced the inflammatory response of ASCs

**Fig. 4:**
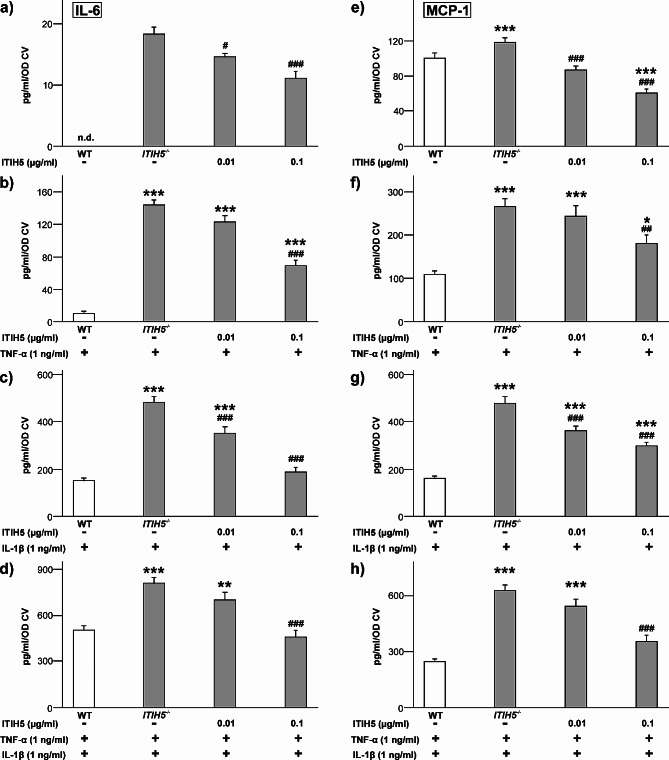
Effect of genetic deletion of ITIH5 (*ITIH5*^*−/−*^) on cytokine release when undifferentiated ASCs were exposed to inflammatory mediators. Cells from wildtype (WT) and knockout mice (*n* = 5 animals each) were either left untreated (-), or stimulated with 1 ng/ml TNF-α or IL-1β, and rITIH5 protein (at 0.01–0.1 µg/ml, as indicated). After 48 h, media were collected to determine cytokine secretion by sandwich ELISA: IL-6 (a-d), MCP-1 (e-h). The data were calculated per cell number (divided by OD CV), and are presented as mean values (+ SEM). The number of experiments was: *n* = 15. Statistical analyses were performed by one-way ANOVA followed by Tukey’s post-hoc test; **p < 0.01, and ***p < 0.001 vs. WT; ^##^*p* < 0.01 and ^###^*p* < 0.001 vs. *ITIH5*^*−/−*^

In an artificial inflammatory environment simulated by TNF-α and IL-1β exposure, ASCs released increased amounts of IL-6 and MCP-1 (Fig. 4). In this environment, the secretory activity of the *ITIH5*^*−/−*^ cells was significantly greater than that of the WT ASCs, while the application of the rITIH5 protein inhibited the knockout effect. When the cells were not stimulated, ANOVA revealed significant differences in the concentrations of IL-6 (F(3;62) = 12,296, *p* < 0,001; Fig. 4a) and MCP-1 (F(3;96) = 19.784; *p* < 0.001; Fig. 4e). Pairwise comparisons revealed that the rITIH5 protein significantly reduced the release of IL-6 and MCP-1 in the *ITIH5*^*−/−*^ cells (*p* < 0.001, d > 0.75). The same was true for stimulation with TNF-α (Fig. 4b&f), with IL-1β (Fig. 4c&g), or when stimulated with both factors (Fig. 4d&h). In the latter case, 0.1 µg/ml rITIH5 protein compensated for the knockout effect by downregulating the release of IL-6 and MCP-1 to the level of that in WT cells.

## Discussion

The technological advances made by mankind over the last 50 years and the associated oversupply of food with a simultaneous reduction in physical activity have made obesity and associated metabolic diseases a global health problem [30]. Obesity is characterized by adipose tissue dysregulation, i.e., an increase in fat tissue size due to an increase in adipocyte size (hypertrophy) and number (hyperplasia), leading to a chronic pro-inflammatory status and presenting an insulin-resistant environment that contributes to type 2 diabetes mellitus [31, 32]. Therefore, understanding the complex processes underlying adipose tissue homeostasis and investigating the factors that lead to its dysfunction is crucial for the development of effective therapeutic strategies to combat this metabolic disease [33].

Adipokines have been identified as key regulators of adipose tissue physiology and development, expanding the understanding of the complex interplay between adipocytes, adipose stem cells, immune cells, and various signaling pathways. Some adipokines have been associated with anti-proliferative activity, immunosuppressive properties, or to inhibit adipogenesis. For example, vaspin (serpin A-12) is an adipokine known for its anti-inflammatory properties by inhibiting the release of proinflammatory cytokines in adipocytes [34, 35]. Chemerin regulates adipocyte differentiation and its knockdown decreases the expression of key genes that regulate glucose and lipid balance and alters metabolic functions in mature adipocytes [36]. It reduces the expression of adipogenic genes and promotes a more fibrotic phenotype of adipose tissue, which is less conducive to fat storage. Adiponectin is known for its proliferation-inhibiting effects on various cell types, including adipocytes, via different signaling pathways. Adiponectin is also associated with improved insulin sensitivity and reduced inflammation [4, 37]. However, adipokines not only act in the periphery, e.g., adipose tissue, but also influence central mechanisms related to hunger, food seeking and food intake. Leptin is a well-known adipokine, that was discovered in 1994. It is predominantly secreted by adipocytes and regulates appetite by signaling satiety in mammals [38–40].

We recently investigated the expression of ITIH5 in human adipose tissue and confirmed the findings of Anveden et al. [24] and Ronn et al. [41] that the concentration of ITIH5 is positively correlated with the patient`s BMI and tissue inflammation status [7]. Based on these findings, ITIH5 was originally proposed as a novel marker of obesity [42]. Interestingly, studies on the adipose tissue of obese non-human primates (*Macaca fascicularis*) also revealed the upregulation of *ITIH5* mRNA expression, confirming its evolutionary conservation [43]. However, the present in vivo study showed that ITIH5 is not an inert indicator of adipose tissue development. Although there was no significant difference in bodyweight or absolute fat mass, the *ITIH5*^*−/−*^ animals exhibited greater adipose tissue weight relative to total bodyweight. In this context, we would like to point out the young age of the experimental animals, which could explain the contradictory result that the relative fat mass increased, although the total bodyweight did not differ between the WT and knockout animals. As in other mammalian organisms, precocious mice (< 3 months) do not accumulate large fat depots on a normal diet. In mice, bodyweight and adipocyte size increase the most during maturation, while total body fat reaches its maximum at middle age, i.e., at 10–14 months [44, 45]. Therefore, the *ITIH5*-knockout effect on the increased development of adipose tissue possibly increases with the age of the experimental animals. On the other hand, we can only speculate why there was no difference in total weight even though fat mass increased. ASCs belong to the family of mesenchymal stem cells (MSCs), which also include bone marrow (bm) stem cells; thus, they share several characteristics, including the ability to undergo multi-differentiation along the mesodermal lineage [46, 47]. Physiological bone growth relies on the bm-MSCs, which develop into bone forming cells or osteoblasts [48]. Theoretically, there is an inverse relationship between the adipogenic and osteogenic differentiation of MSCs, meaning that development into an adipocyte occurs at the expense of an osteoblastic phenotype and vice versa [49]. Since adipogenesis appeared to be increased in the *ITIH5*^*−/−*^ mice, it is possible that this increase was accompanied by a loss in bone mass, which would compensate for their body weight being similar to that of the WT mice. In other words, if the same *ITIH5*-knockout effect found for ASCs occurs also in bm-MSCs, namely, increased adipogenic differentiation, then bone mass would decrease, as would the bodyweight of the animals, because adipocytes have a lower weight than bone cells [50].

Nevertheless, genetic deletion of *ITIH5* led to an increase in fat mass, supporting our hypothesis that ITIH5 acts a as negative regulator of adipose tissue expansion. Subsequent in vitro experiments with isolated ASCs also confirmed the in vivo results. Unfortunately, we only used adipose tissue explants solely for the isolation of ASCs and did not histologically examine the adipose tissue from WT and *ITIH5*^*−/−*^ mice. This represents a certain limitation of the present study, as these analyses would have allowed us to determine whether the differences in adipose tissue expansion depended on adipocyte hyperplasia or hypertrophy. With respect to the cell culture experiments, we must conclude that both processes may have contributed to the differences in adipose tissue expansion between the WT and knockout animals. Both proliferation and adipogenic differentiation were greater in ASCs from the *ITIH5*^*−/−*^ compared to WT animals. According to the in vivo findings, the number and size of ASCs may have been greater in the adipose tissue of knockout mice. Interestingly, the application of the rITIH5 protein resulted in a concentration-dependent reduction in proliferation and adipogenic differentiation, demonstrating that exogenous ITIH5 can effectively compensate for genetic deletion and restore cell numbers to levels similar to those of WT cells. These results emphasize the potential of the ITIH5 protein as a modulator of adipocyte precursor cell proliferation and differentiation. The potent effect of the rITIH5 protein on ASCs underscores its potential as a therapeutic agent (“biological”) to correct dysregulations in human adipose tissue that are often associated with obesity and metabolic disorders. In recent years, several peptides and other bioengineered products have emerged as promising candidates for treating obesity [51]. Semaglutide, a GLP-1 receptor agonist, is certainly the best-known modified peptide currently used to treat obesity [52]. It is not yet clear exactly which amino acid regions of the ITIH5 protein mediate the effects induced in ASCs. Therefore, to develop a suitable biological drug for this field of application in the future, the ITIH5 protein may still need to be truncated, modified or stabilized, as has also been proposed for its possible use in personalized cancer therapy [53].

ASCs from *ITIH5*^*−/−*^ mice did not differ from those from WT mice in terms of osteogenic differentiation, whereas knockout ASCs showed a greater capacity to differentiate into the chondrogenic lineage, which could be neutralized or even reversed by the application of the rITIH5 protein. This result corresponds well with our findings on human ASCs, in which the application of the rITIH5 protein also inhibited the chondrogenic differentiation [7]. In particular, ITIH5 modulates the signaling pathways of cytokines belonging to the large transforming growth factor (TGF)-β superfamily [15], which are, for example, crucial for the differentiation of human fibroblasts into myofibroblasts [54]. Thus, the knockout of *ITIH5* may have upregulated the expression of TGF-β superfamily targets in ASCs, e.g., BMP-2 and − 4, which in turn increased adipogenesis. Chondrogenic and osteogenic differentiation in ASCs is also driven by members of the TGF-β superfamily, i.e., TGF-β3, BMP-6, -7 and − 14 [55–59]. However, since adipogenic and chondrogenic differentiation was primarily affected by the *ITIH5*-knockout, the interaction between ITIH5 and TGF-β appears to primarily modulate soft-tissue development in ASCs.

Finally, we examined the secretion of the cytokines IL-6 and MCP-1 in response to stimulation with inflammatory mediators. Compared with those from WT mice, ASCs from *ITIH5*^*−/−*^ mice released more of these cytokines. However, we can only present and discuss the in vitro data because we did not measure the levels of inflammatory markers in the adipose tissue of the experimental animals. Hence, we cannot determine whether ITIH5-knockout led to an increase in adipose tissue inflammation, which represents a further limitation of the present study.

The application of the rITIH5 protein effectively reduced this pro-inflammatory response by decreasing the levels of IL-6 and MCP-1. These results are consistent with our findings on human ASCs in which the rITIH5 protein has the same effects [7], and they emphasize the immunosuppressive properties of ITIH5 and its potential role in regulating inflammation in the adipose tissue microenvironment. This property is particularly important because increased adipogenesis in individuals with obesity is closely associated with a systemic inflammatory state mediated primarily by increased secretion of pro-inflammatory cytokines [60]. However, in adipose tissue, ASCs and adipocytes are the source of IL-6 and MCP-1, whereas M1 macrophages primarily release IL-1β and TNF-α [28]. Future experiments should therefore investigate whether the rITIH5 protein also modulates the secretory activity of these immune cells to confirm its immunosuppressive effect.

## Conclusions

**Fig. 5:**
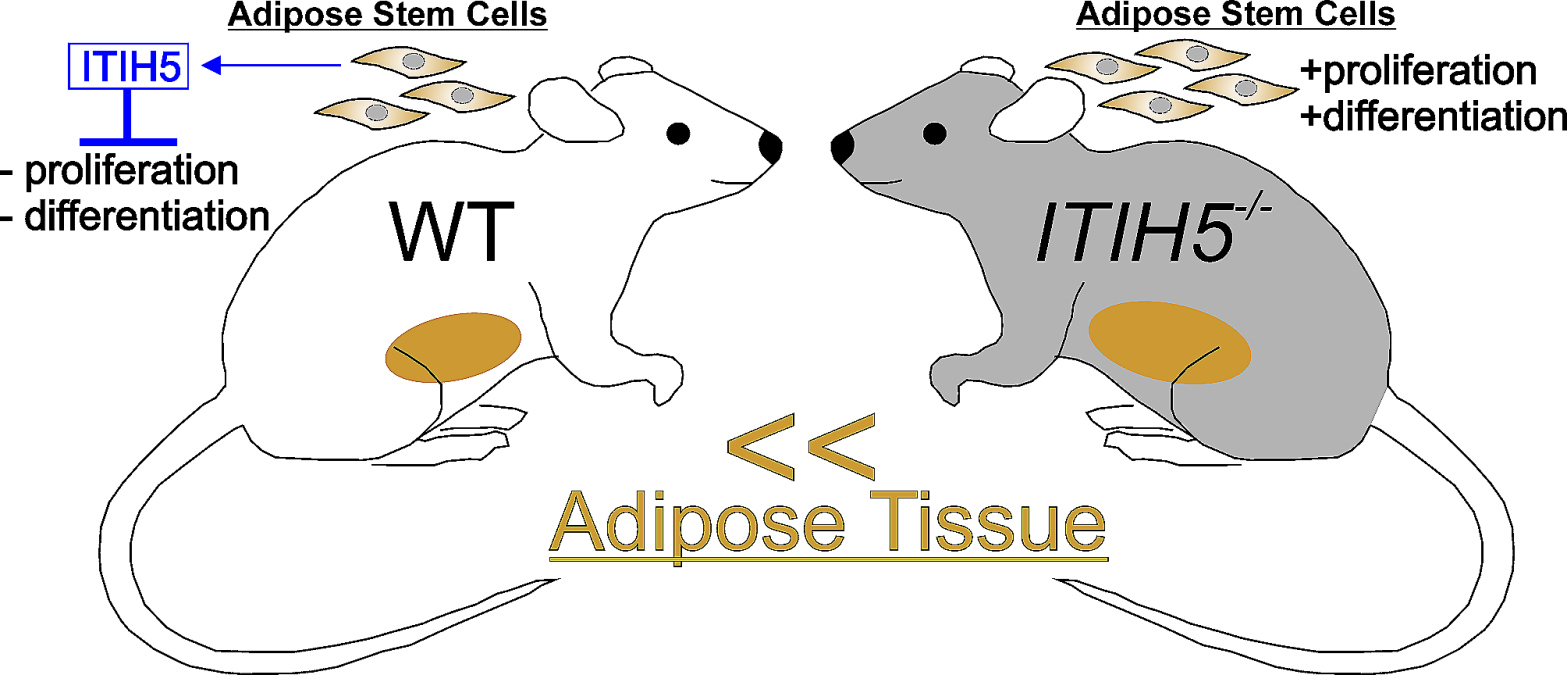
Genetic deletion of *ITIH5* increases the proliferation and adipogenic differentiation of adipose stem cells (ASCs) compared to cells from wildtype mice expressing the native ITIH5 protein. Adipose tissue development is characterized by an increase in the number of mature adipocytes that arise from proliferating ASCs (hyperplasia), and by enhanced accumulation of lipid deposits during adipogenic differentiation (hypertrophy). Thus, both processes could be the reason for the increased adipose tissue development in the *ITIH5*^*−/−*^ mice. Moreover, both the proliferation and the adipogenic differentiation of ASCs are inhibited by the ITIH5 protein (native and recombinant), suggesting that the ITIH5 molecule is a negative regulator of adipose tissue development

In summary, our study provides novel insights into the function of ITIH5 in mammalian adipose tissue. Genetic deletion of *ITIH5* resulted in an increase in adipose tissue mass, which can be explained by increased proliferation and adipogenic differentiation of ASCs and is summarized in Fig. 5. The application of the rITIH5 protein reversed the observed knockout effects, emphasizing the role of ITIH5 as a negative regulator of adipose tissue development. In addition, ITIH5 reduced the release of pro-inflammatory cytokines by ASCs, which may be a particularly important function of ITIH5 in the context of increased inflammation during unrestrained adipose tissue expansion. Overall, our results support the notion that ITIH5 is a paracrine-acting adipokine that maintains adipose tissue homeostasis.

### Electronic supplementary material

Below is the link to the electronic supplementary material.

Supplementary Material 1

## Data Availability

All data sets used and/or analyzed during the current study are available from the corresponding author on reasonable request.

## Abbreviations

ASCs: Adipose stem cells
bFGF: Basic fibroblast growth factor
bm: Bone marrow
BMI: Body mass index
BMP: Bone morphogenetic protein
CMKLR1: Chemerin chemokine-like receptor 1
CV: Crystal violet
ECM: Extracellular matrix
IL-6/-1β: Interleukin-6/-1β
ITIH5: Inter-α-trypsin inhibitor heavy chain 5
MCP-1: Monocyte chemoattractant protein-1
MSC: Mesenchymal stem cells
SEM: Standard error of the mean
TNF-α: Tumor necrosis factor
TGF-β: Transforming growth factor
r: recombinant
WT: Wildtype
