# Supplementary material for: Genetic deletion of ITIH5 leads to increased development of adipose tissue in mice
Source: Biol Res. 2024 Aug 29;57:58. doi: 10.1186/s40659-024-00530-0 (PMC11360682; doi:10.1186/s40659-024-00530-0)
Supplement: Supplementary file 1 — Supplementary Material 1 [file 40659_2024_530_MOESM1_ESM.pdf]

iLAS

Formblatt: FB-VTK-TS-001-03
Anzeige der Tötung von Tieren zu wissenschaftlichen Zwecken (A4 Anzeige)

An die/den
Tierschutzbeauftragte/n der RWTH Aachen
Universitätsklinikum der RWTH Aachen
Institut für Versuchstierkunde
Pauwelsstr. 30
52074 Aachen

Begründung für das Töten von Wirbeltieren zu wissenschaftlichen Zwecken (Forschung) gem. § 4 Abs. 3 des Tierschutzgesetzes vom 18. Mai 2006 I 1206, 1313 zuletzt geändert durch Art 3 des Gesetzes vom 28. Juli 2014 BGBl. I S. 1308

Bei der Tötung von Wirbeltieren zu wissenschaftlichen Zwecken ist auch außerhalb von anzeige- oder genehmigungspflichtigen Tierversuchen der Tierschutzbeauftragte mit einzubeziehen. Auf dieser Grundlage teile ich der/dem Tierschutzbeauftragten der RWTH mit:

Abteilung

Institut / Klinik / Abteilung	Pathologie

Leiter

Titel / Vor- und Zuname	Prof. Dr. Edgar Dahl
Institut / Klinik / Abteilung	Pathologie, Molekulare Onkologie
Telefonnummer / Funkruf	88431
E-Mail Adresse	edahl@ukaachen.de

Personen, die die Tötung vornehmen (sollen mehr als 3 Personen die Tötung vornehmen können, verwenden Sie bitte das Formular in der Anlage dieses Dokuments)

Titel / Vor- und Zuname	M. Sc. Sophia Villwock		
Institut / Klinik / Abteilung	Pathologie, Molekulare Onkologie		
Telefonnummer / Funkruf	89164		
E-Mail Adresse	svillwock@ukaachen.de		
Sachkunde (§ 2 u. Anl. 1 Abs. 2 TierSchVersV)<br>*Bitte fügen Sie entsprechende Bescheinigungen hinzu, wenn uns diese nicht vorliegen.*	Felasa B Kurs	☐ nein<br>☒ ja	Aachen, 14.01.-25.01.2019<br>Ort u. Datum des Kurses
	AFW Kurs	☐ nein<br>☐ ja	Ort u. Datum des Kurses
	Sonstige	☐ nein<br>☐ ja	Ort u. Datum der Fortbildung

Personen, die die Tötung vornehmen

Titel / Vor- und Zuname	Klicken Sie hier, um Text einzugeben.		
Institut / Klinik / Abteilung	Klicken Sie hier, um Text einzugeben.		
Telefonnummer / Funkruf	Klicken Sie hier, um Text einzugeben.		
E-Mail Adresse	Klicken Sie hier, um Text einzugeben.		
Sachkunde (§ 2 u. Anl. 1 Abs. 2 TierSchVersV)<br>*Bitte fügen Sie entsprechende Bescheinigungen hinzu, wenn uns diese nicht vorliegen.*	Felasa B Kurs	☐ nein<br>☐ ja	Ort u. Datum des Kurses
	AFW Kurs	☐ nein<br>☐ ja	Ort u. Datum des Kurses
	Sonstige	☐ nein<br>☐ ja	Ort u. Datum der Fortbildung

Titel / Vor- und Zuname	Klicken Sie hier, um Text einzugeben.		
Institut / Klinik / Abteilung	Klicken Sie hier, um Text einzugeben.		
Telefonnummer / Funkruf	Klicken Sie hier, um Text einzugeben.		
E-Mail Adresse	Klicken Sie hier, um Text einzugeben.		
Sachkunde (§ 2 u. Anl. 1 Abs. 2 TierSchVersV)<br>*Bitte fügen Sie entsprechende Bescheinigungen hinzu, wenn uns diese nicht vorliegen.*	Felasa B Kurs	☐ nein<br>☐ ja	Ort u. Datum des Kurses
	AFW Kurs	☐ nein<br>☐ ja	Ort u. Datum des Kurses
	Sonstige	☐ nein<br>☐ ja	Ort u. Datum der Fortbildung

Bezeichnung des Versuchsvorhabens

Bitte geben Sie den Titel und eine kurze Beschreibung des Versuchsvorhabens an.

Aufschlüsselung von weiteren zelltypspezifischen ITIH5 Funktionen in einem ITIH5 kockout Mausmodell

Angaben zu den Versuchstieren (sollen mehr als 8 Stämme / Linien zu wissenschaftlichen Zwecken getötet werden, verwenden Sie bitte das Formular in der Anlage dieses Dokuments)

Tierart *Maus*

Stamm/Linie

Bezeichnung	***belastet***		***wenn belastet: Az der genehmigten Zucht***	***Tierbase-Nr.***	***Anzahl der Tiere***
	ja	***nein***			
BALB/c-Itih5tm2350Arte	☐	☒		*920*	*500*
BALB/cAnNRj	☐	☒			*100*
Klicken Sie hier, um Text einzugeben.	☐	☐			
Klicken Sie hier, um Text einzugeben.	☐	☐			
Klicken Sie hier, um Text einzugeben.	☐	☐			
Klicken Sie hier, um Text einzugeben.	☐	☐			
Klicken Sie hier, um Text einzugeben.	☐	☐			
Klicken Sie hier, um Text einzugeben.	☐	☐			
				Σ	600

Geschlecht ☒ *männlich* ☒ *weiblich*

Gewicht *10-30 g*

Alter *5 Wochen aufwärts*

Auch bei der Tötung von Tieren zu wissenschaftlichen Zwecken ist die Zahl der beantragten Tiere durch eine Biometrie bzw. Abschätzung der Tierzahlen aus der Erfahrung zu begründen. Aus Erfahrung, der Literatur und/ oder einer Stichprobengrößenberechnung wird voraussichtlich die unten angegebene Anzahl von Tieren benötigt:

500

Herkunft der Tiere

☒ Es handelt sich um eigens für Tierversuche gezüchtete Tiere.

☐ Die Tiere sind nicht zu Versuchszwecken gezüchtet worden.

☐ Im Falle von Hunden, Katzen, Halbaffen oder Affen, die nicht eigens für Tierversuche gezüchtet wurden, wurde **vor** der Tötung eine Ausnahmegenehmigung bei der zuständigen Behörde beantragt. Die erteilte Ausnahmegenehmigung liegt dieser

Anzeige bei.

Vernünftiger Grund (§§ 1 , 17 TierSchG) / Stand der wissenschaftlichen Erkenntnisse (§ 7a Abs. 2 Nr. 1 TierSchG)

Angabe des vernünftigen Grundes zur Tötung eines Wirbeltieres (§§ 1 , 17), insbesondere Erläuterung der Notwendigkeit für die Tötung der Wirbeltiere unter Berücksichtigung des jeweiligen Standes der wissenschaftlichen Erkenntnisse (§ 7a Abs. 2 Nr. 1 TierSchG).

Die Funktion von Inter-alpha-Trypsin Inhibitor 5 (ITIH5) wurde in verschiedenen Tumorentitäten wie Darm-, Blasen-, Lungen-, Pankreas-, und Brustkrebs als tumorsuppressiv beschrieben. Weiterhin spielt ITIH5 eine Rolle in Entzündungsprozessen wie Pankreatitis, Kolitis und Sepsis. In vorhergehenden Untersuchungen unserer Forschungsgruppe wurde anhand der ITIH5 knockout Mauslinie bereits eine schwache phänotypische Veränderung der Haut-Epidermis gefunden. Dort wurde ITIH5 als mögliches regulatorisches Molekül identifiziert, dessen Verlust möglicherweise eine Störung der natürlichen Hautbarrierefunktion bedingen könnte. Daneben wurde in den knockout Mäusen phänotypisch lediglich ein tendenziell etwas verringertes Körpergewicht und einzelne Auffälligkeiten in Blut- und Serumparametern im Vergleich zu den ITIH5 positiven Kontrollmäusen gefunden. Weitere zelltypspezifische Funktionen von ITIH5 wurden bisher noch nicht untersucht und sind noch unbekannt. Daher sollen anhand des knockout Modells weitere Untersuchungen vorgenommen werden. Darunter fällt zum Beispiel eine genauere Untersuchung der ITIH5 Funktion in immunologischen und entzündlichen Prozessen sowie in neurologischen Prozessen.

Literatur:

1. Kloten, V. et al. Epigenetic inactivation of the novel candidate tumor suppressor gene ITIH5 in colon cancer predicts unfavorable overall survival in the CpG island methylator phenotype Epigenetic inactivation of the novel candidate tumor suppressor gene ITIH5 in colon can. Epigenetics 9, 1290–1301 (2014).
2. Rose, M. et al. Epigenetic inactivation of ITIH5 promotes bladder cancer progression and predicts early relapse of pT1 high-grade urothelial tumours. Carcinogenesis 35, 727–736 (2014).
3. Dötsch, M. M. et al. Low expression of ITIH5 in adenocarcinoma of the lung is associated with unfavorable patients' outcome. Epigenetics 10, 903–912 (2015).
4. Sasaki, K. et al. Genome-wide in vivo RNAi screen identifies ITIH5 as a metastasis suppressor in pancreatic cancer. Clin. Exp. Metastasis 34, 229–239 (2017).
5. Veeck, J. et al. The extracellular matrix protein ITIH5 is a novel prognostic marker in invasive node-negative breast cancer and its aberrant expression is caused by promoter hypermethylation. Oncogene 27, 865–876 (2008).

6. Huth, S. et al. Inter-α-trypsin inhibitor heavy chain 5 (ITIH5) is overexpressed in inflammatory skin diseases and affects epidermal morphology in constitutive knockout mice and murine 3D skin models. Exp. Dermatol. 24, 663–668 (2015).
7. Walley, A. J. et al. ITIH-5 Expression in Human Adipose Tissue Is Increased in Obesity. Obesity 20, 708–714 (2011).

Tötungsmethode (§ 2 Abs. 2 TierSchVersV)

Angabe der Tötungsmethode.

CAVE: Falls eine andere Tötungsmethode als in Anlage 2 TierSchVersV (Gesetzestext siehe Anhang) vorgesehen, angewandt werden soll, muss eine behördliche Genehmigung eingeholt werden.

Cervicale Dislokation

Ort, Beginn und voraussichtliche **Dauer** des Versuchsvorhabens:

Ort	RWTH Aachen Uniklinik, Pauwelsstraße 30, 52074 Aachen, Institut für Versuchstierkunde
Beginn	Revitalisierung Januar 2019
Dauer<br>*(max. 3 Jahre)*	3 Jahre

Angaben gemäß Versuchstiermeldeverordnung (VersTierMeldV)

Verwendungszweck

Bitte hier mit Code eintragen (Code siehe in der Anlage dieses Dokuments)

PB1, PB3, PB7, PB10, PT21

Falls Sie „Andere“ (PB13, PT32, PR53, PR64, PR71, PR82, PR103, PR106) angeben, beschreiben Sie den Verwendungszweck hier genauer:

Gesetzlich vorgeschriebene Überprüfung nach…

Folgenden Code nur angeben, wenn bei „Verwendungszweck“ eine der Optionen PR51 bis PR106 (Regulatorischer Zweck,…) angegeben wurde. Die Rechtsvorschrift muss entsprechend dem vorgesehenen Hauptverwendungszweck angegeben werden.

Bitte hier mit Code eintragen (Code siehe in der Anlage dieses Dokuments)

Falls Sie „Andere“ (LT10) angeben, ist die spezifische Rechtsvorschrift, die der Verwendung zugrunde liegt, zu benennen:

Rechtsquelle

Folgendes nur ankreuzen, wenn eine Rechtsvorschrift angegeben wurde.

Ausschlaggebend ist dabei nicht, wer die Prüfung in Auftrag gibt, sondern welchen Vorschriften nachgekommen wird, wobei der weitreichenderen Vorschrift Vorrang eingeräumt wird. Dienen die nationalen Vorschriften der Umsetzung von EU-Recht (z.B. Umsetzung von Richtlinien der EU, Durchführung von Verordnungen der EU u. Ä.), muss „Vorschriften, die EU-Anforderungen erfüllen" gewählt werden.

☐ **(LO1)** Vorschriften, die EU-Anforderungen erfüllen

☐ **(LO2)** Vorschriften, die nur nationale Anforderungen erfüllen

☐ **(LO3)** Vorschriften, die EU-externe Anforderungen erfüllen

05.11.19

(Datum / Unterschrift des Leiters)

Diese Begründung muss vor Beginn des Versuchsvorhabens ausgefüllt und unterschrieben im Institut für Versuchstierkunde, bei der/dem Tierschutzbeauftragten abgegeben werden.
